# Treatment of diabetic mice with the SGLT2 inhibitor TA-1887 antagonizes diabetic cachexia and decreases mortality

**DOI:** 10.1038/s41514-017-0012-0

**Published:** 2017-09-08

**Authors:** Taichi Sugizaki, Shunshun Zhu, Ge Guo, Akiko Matsumoto, Jiabin Zhao, Motoyoshi Endo, Haruki Horiguchi, Jun Morinaga, Zhe Tian, Tsuyoshi Kadomatsu, Keishi Miyata, Hiroshi Itoh, Yuichi Oike

**Affiliations:** 1 Department of Molecular Genetics, Graduate School of Medical Sciences, Institute of Resource Development and Analysis, Kumamoto University, 1-1-1 Honjo, Chuo-ku, Kumamoto, 860-8556 Japan; 2 Department of Immunology, Allergy and Vascular Medicine, Graduate School of Medical Sciences, Institute of Resource Development and Analysis, Kumamoto University, 1-1-1 Honjo,Chuo-ku, Kumamoto, 860-8556 Japan; 3 Division of Endocrinology, Metabolism and Nephrology, Department of Internal Medicine, School of Medicine, Keio University, 35 Shinanomachi, Shinjuku-ku, Tokyo, 160-8582 Japan

## Abstract

A favorable effect of an inhibitor of the sodium–glucose cotransporter 2 (SGLT2i) on mortality of diabetic patients was recently reported, although mechanisms underlying that effect remained unclear. Here, we examine SGLT2i effects on survival of diabetic mice and assess factors underlying these outcomes. To examine SGLT2i treatment effects in a model of severe diabetes, we fed genetically diabetic *db/db* mice a high-fat diet and then assessed outcomes including diabetic complications between SGLT2i TA-1887-treated and control mice. We also compare effects of SGLT2i TA-1887 with those of lowering blood glucose levels via insulin treatment. Untreated *db/db* mice showed remarkable weight loss, or cachexia, while TA-1887-treated mice did not but rather continued to gain weight at later time points and decreased mortality. TA-1887 treatment prevented pancreatic beta cell death, enhanced preservation of beta cell mass and endogenous insulin secretion, and increased insulin sensitivity. Moreover, TA-1887 treatment attenuated inflammation, oxidative stress, and cellular senescence, especially in visceral white adipose tissue, and antagonized endothelial dysfunction. Insulin treatment of *db/db* mice also prevented weight loss and antagonized inflammation and oxidative stress. However, insulin treatment had less potent effects on survival and prevention of cellular senescence and endothelial dysfunction than did TA-1887 treatment. SGLT2i treatment prevents diabetic cachexia and death by preserving function of beta cells and insulin target organs and attenuating complications. SGLT2i treatment may be a promising therapeutic strategy for type 2 diabetes patients with morbid obesity and severe insulin resistance.

## Metabolic syndrome: SGLT2i prevents diabetic cachexia and prolongs survival

Sodium-glucose cotransporter 2 inhibitor (SGLT2i) has a favorable effect on mortality of diabetic subjects, but the mechanism stays unclear. Taichi Sugizaki at Kumamoto University examined SGLT2i effects in severe diabetic obese mice, and discovered that they showed prolonged survival without pathological weight loss, or cachexia. As with SGLT2i, Insulin also prevented cachexia, improved pancreatic beta cell function, insulin sensitivity and some organ damages. However, what makes SGLT2i important was to suppress cellular aging or vessel inflammation, while insulin accelerated those developments, which may lead to a result that SGLT2i has contributed to prolonged survival more than insulin. SGLT2i demonstrates an association with survival period upon maintaining good condition of pancreatic beta cells and insulin target organs, providing insight into strategies for treatment of severe diabetes.

## Introduction

Type 2 diabetes incidence is increasing worldwide and is a primary cause of death. Along with hypertension and dyslipidemia, type 2 diabetes is an important risk factor for cardiovascular disease and is accompanied by microvascular complications; thus prevention of macrovascular and microvascular complications is a critical issue in diabetes treatment.^1, 2^ Large-scale studies have been carried out relevant to prevention of microvascular complications, but thus far, only a few trials of antidiabetic agents have demonstrated improvement of cardiovascular events and decreased mortality.^3–6^ The UK Prospective Diabetes Study Group showed that metformin treatment of overweight patients decreased diabetes-related mortality, while intensive blood-glucose control through antidiabetic agents, including insulin, did not significantly reduce cardiovascular events but tended to decrease myocardial infarction, which included non-fatal and fatal myocardial infarction and sudden death.^3, 4^ However, in a 10-year post-interventional follow-up (UKPDS 80), post-trial risk reductions emerged in the intensive therapy group for diabetes-related death, myocardial infarction and death from any cause, suggesting that improvements in controlling blood glucose levels are crucial to manage cardiovascular events and decrease mortality.

Relevant to this need, the EMPA-REG OUTCOME trial showed a significant effect of the sodium–glucose cotransporter 2 inhibitor (SGLT2i) empagliflozin in antagonizing death from cardiovascular causes or death from any cause in patients with type 2 diabetes at high cardiovascular risk.^7^ In this trial, however, there were no significant between-group differences in rates of myocardial infarction or stroke. Interpretations of this outcome vary, but some propose that factors other than those that decrease blood glucose levels contribute to decreased mortality from cardiovascular or other causes.^8–10^

Here, to test effects of an SGLT2i in a severe diabetic mouse model, we employed genetically diabetic *db/db* mice fed a high-fat diet (HF). We compared treatment outcomes including diabetic complications between mice treated with the SGLT2i TA-1887 and untreated controls and also assessed outcomes following insulin treatment. We confirm that SGLT2i treatment has beneficial effects in improving diabetic outcomes relative to insulin treatment and discuss mechanisms potentially underlying these effects.

## Results

### TA-1887 treatment decreases mortality in severely diabetic mice

**Fig. 1:**
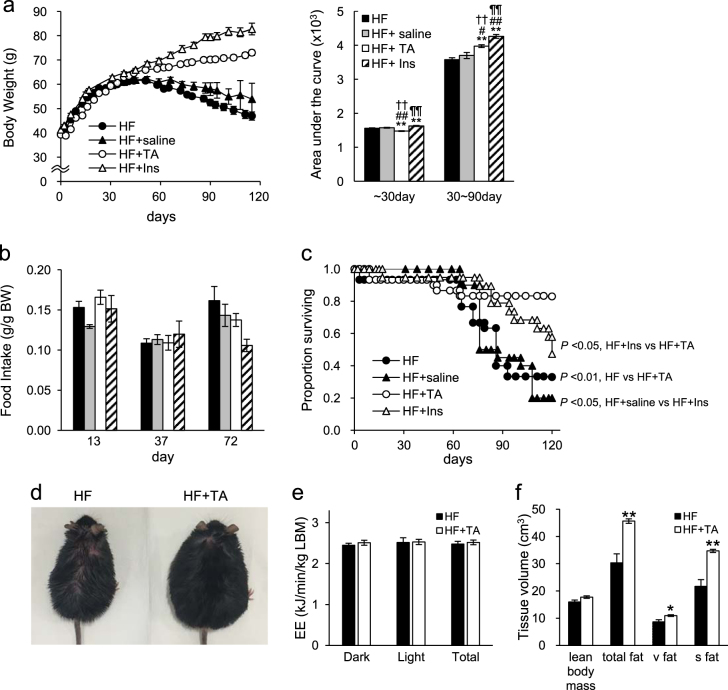
TA-1887-treated or insulin-treated severely diabetic mice show enhanced survival and increased body weight but does not alter food intake or energy expenditure. **a** left: Changes in body weight in *db/db* mice fed a high-fat diet (HF) or comparable mice treated with saline (HF + saline) or with TA-1887(HF + TA) or insulin (HF + Ins) (*n* = 20–30). right: AUC for body weight at indicated periods. **b** Food intake of each group at 13, 37, and 72 days (*n* = 6). **c** Proportion of animals surviving 4 months (*n* = 20–30). **d** Representative appearance of mice in indicated groups at day 80. **e** Energy expenditure (EE) after 9 weeks of each treatment, as determined by indirect calorimetry (*n* = 6). **f** Volume of lean body mass, total fat, visceral fat (v fat) and subcutaneous fat (s fat), as measured by computed tomography (CT) after 3 months of each treatment (*n* = 6). Values shown are means ± SEM. *p < 0.05, **p < 0.01 versus HF, and ^#^ *p* < 0.05, ^##^ *p* < 0.01 versus HF + saline, and ^¶¶^ *p* < 0.01 versus HF + TA and ^††^ *p* < 0.01 versus HF + Ins

For analysis, we used TA-1887, an SGLT2i with selectivity for SGLT2 versus SGLT1, similar to canagliflozin.^11, 12^ To determine treatment effects, we evaluated *db/db* mice (also known as *Lepr* −/− mice) fed a HF diet as a model of severe diabetes and treated them with or without TA-1887. As reported by others,^13, 14^ in 1st month body weight of TA-1887-treated mice decreased relative to that of untreated mice (Fig. 1a). However, after a month, untreated mice showed first a slow increase in body weight and then a decline, whereas body weight of TA-1887-treated mice remained greater overall than that of untreated animals (Fig. 1a). As a comparison, mice treated with insulin showed continued weight gain, an effect not seen in saline-injected controls (Fig. 1a). Although TA-1887 or insulin treatment increased body weight, insulin-treated mice showed enhanced weight gain relative to TA-1887 animals (Fig. 1a). We observed no difference in food intake between TA-1887 and insulin-treated groups (Fig. 1b), suggesting that body weight differences between groups could be due to differences in lipid accumulation.

*db/db* mice fed a HF diet normally survive for only 3–5 months, while those fed normal chow live approximately 10 months.^15, 16^ Either TA-1887 or insulin treatment significantly increased survival of *db/db* mice fed a HF diet, although survival rates of TA-1887-treated mice were significantly greater (Fig. 1c).

Following the death of treated or untreated mice, we performed X-ray computed tomography (CT) scanning and necropsy (Supplementary Table 3). Our investigation included evaluation of potential brain hemorrhage, cerebral infarction, vascular calcification, vascular obstruction, and myocardial infarction. We also searched for cancerous masses in lung, liver, stomach, intestine, and kidney. We found none of these pathologies (data not shown) and were therefore unable to determine the cause of death of any of these mice. Most untreated mice, however, lost more than 10% of body weight before death, an outcome rarely seen in TA-1887-treated or insulin-treated mice (Supplementary Table 3). These observations suggest that untreated mice die from events associated with diabetic cachexia, a condition not suffered by treated mice.

### TA-1887 treatment does not alter energy expenditure in *db/db* mice fed a HF diet

By approximately day 80 of drug treatment, untreated mice showed thinning coat fur (Fig. 1d), while TA-1887-treated mice appeared healthy but severely obese. Given differences in body weight between groups, we measured energy expenditure after 9 weeks of drug treatment in TA-1887-treated and untreated groups by indirect calorimetry and observed no differences between groups (based on lean body mass) (Fig. 1e). To analyze potential changes in tissue composition accompanying weight changes, we performed CT scanning after 3 months of drug treatment. Adipose tissue volume in TA-1887-treated mice significantly increased relative to untreated controls, particularly in subcutaneous adipose tissue, although lean body mass was comparable between groups (Fig. 1f).

### TA-1887 antagonizes hyperglycemia and increases endogenous insulin secretion

**Fig. 2:**
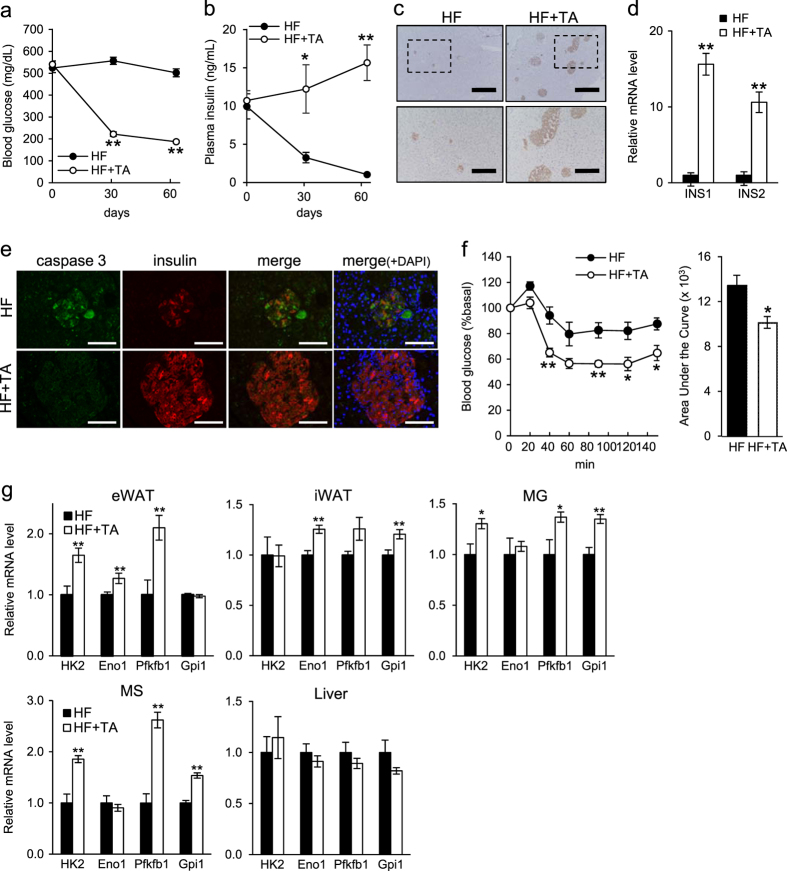
Protective effect of TA-1887 on pancreas and insulin sensitivity in *db/db* mice fed a high-fat (HF) diet. **a**, **b** Blood glucose and plasma insulin (*n* = 8) levels at 0, 1, and 2 months of indicated treatments. **c** Image showing insulin immunostaining in pancreatic tissue of representative mice after 4 months of indicated treatment. Squares in upper panels are magnified in corresponding lower panels. Scale bars: 500 μm (upper), 200 μm (lower). **d** Analysis of *INS1* and *INS2* mRNA expression in mouse pancreatic tissue after 4 months of treatment (*n* = 5–9). **e** Representative double immunostaining of insulin and active caspase 3 in pancreatic tissue after 4 months of treatment. Scale bars: 100 μm. **f** Insulin tolerance test after 10 weeks of drug treatment and corresponding AUC (*n* = 5–9). **g** levels of mRNAs encoding glycolytic enzymes (*n* = 5–9) in eWAT, iWAT, MG, MS and liver after 4 months of treatment. DAPI, 4',6-diamidino-2-phenylindole; INS1, insulin I; INS2, insulin II; HK2, hexokinase 2; Eno1, enolase 1; PFKFB1, 6-phosphofructo-2-kinase/fructose-2,6-biphosphatase 1; Gpi1, glucose phosphate isomerase 1. Values shown are means ± SEM. *p < 0.05 and **p < 0.01 versus HF

We next evaluated blood glucose levels of *db/db* mice fed a HF diet. TA-1887 treatment over a 60-day period markedly reduced blood glucose levels (Fig. 2a). Plasma insulin levels in untreated mice decreased over time but tended to increase in TA-1887-treated mice (Fig. 2b). Immunohistochemical insulin staining in pancreatic tissue revealed an increased volume of pancreatic beta cells (Fig. 2c). Furthermore, real-time polymerase chain reaction (PCR) analysis of genes in pancreas indicated increased levels of the insulin transcripts INS1 and INS2 in TA-1887-treated relative to untreated controls (Fig. 2d). Moreover, to assess pancreatic beta cell death, which could be associated with changes in endogenous insulin secretion, we performed double immunostaining of insulin and the apoptosis marker cleaved (active) caspase 3 in pancreatic tissues. Insulin staining increased but that of cleaved (active) caspase 3 decreased in pancreatic islets of TA-1887-treated relative to untreated mice, suggesting that blocking beta cell death preserves beta cell mass (Fig. 2e).

### TA-1887 treatment enhances insulin sensitivity

Next we assessed insulin sensitivity using an intraperitoneal insulin tolerance test (IPITT). TA-1887-treated *db/db* mice fed a HF diet showed significantly lower blood glucose levels than did untreated controls, indicating improved insulin sensitivity (Fig. 2f). To identify target organs underlying this effect, we evaluated gene expression by real-time PCR. Expression of glycolytic genes increased in epididymal white adipose tissue (eWAT), inguinal WAT (iWAT), gastrocnemius muscle (MG) and soleus muscle (MS) following TA-1887 treatment relative to untreated controls (Fig. 2g). However, expression of glycolytic genes in liver or brown adipose tissue (BAT) was comparable between groups (Fig. 2g and Supplementary Fig. 1a).

### TA-1887 attenuates systemic and tissue inflammation and reduces levels of senescence markers in severely diabetic mice

**Fig. 3:**
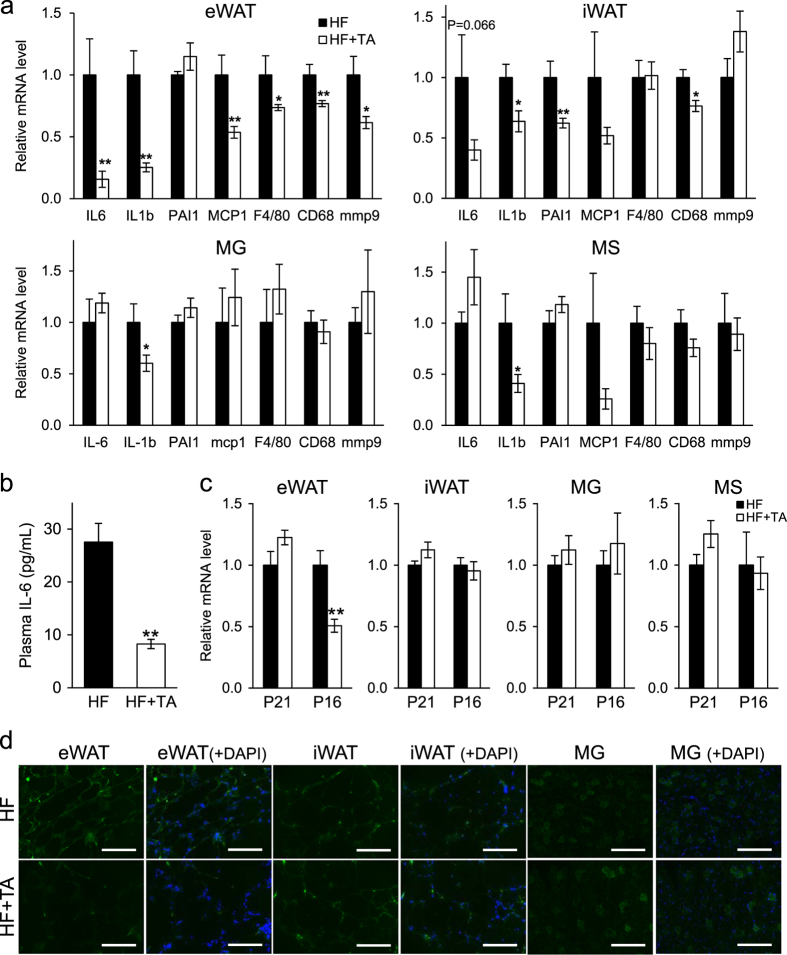
Expression of inflammatory and senescence markers in TA-1887-treated *db/db* mice fed a high-fat diet. **a** mRNA levels of inflammatory mediators in eWAT, iWAT, MG and MS after 4 months of indicated treatment (*n* = 5-9). **b** Plasma IL-6 concentration after 2 months of drug treatment, as determined by ELISA (*n* = 8). **c** mRNA levels of the senescence markers p21 and p16^INK4a^ in eWAT, iWAT, MG and MS after 4 months of indicated treatment (*n* = 5–9). **d** Senescence-associated staining for SPiDER beta-Gal in frozen sections of eWAT, iWAT and MG tissues after 4 months of treatment. Scale bars: 200 μm. IL-6, interleukin-6; IL-1b, interleukin-1b; PAI-1, plasminogen activator inhibitor-1; MCP1, monocyte chemoattractant protein-1; mmp9, matrix metalloproteinase 9. Values shown are means ± SEM. *p < 0.05 and **p < 0.01 versus HF

To indentify factors underlying improved insulin sensitivity and glucose utilization, we evaluated levels of transcripts encoding inflammatory mediators in eWAT, iWAT, MG and MS from *db/db* mice treated and untreated with TA-1887.^17, 18^ Expression of several inflammatory mediators such as IL-6, IL-1b, MCP1, CD68 and mmp12 decreased, particularly in eWAT and iWAT, in TA-1887-treated mice relative to untreated controls, and some reduction in inflammatory markers was seen in MG and MS (Fig. 3a). Levels of inflammatory transcripts were partially reduced in liver (but not in BAT) (Supplementary Fig. 1b). In terms of systemic inflammation, TA-1887 treatment reduced plasma IL-6 levels significantly relative to untreated controls (Fig. 3b), suggesting that levels of inflammatory mediators decrease throughout the body. We also performed immunostaining for Mac-3, a surface glycoprotein that serves as a macrophage marker, to assess macrophage infiltration of eWAT and iWAT. Mice treated with TA-1887 showed decreased Mac-3 staining in both eWAT and iWAT, suggesting that reduced tissue inflammation contributes to reduced plasma IL-6 levels (Supplementary Fig. 2a).

Cellular senescence is associated with inflammation and marked by expression of genes such as p21 and p16^Ink4a^, effectors of cellular aging.^19, 20^ Thus, we assessed p21 and p16^Ink4a^ expression in eWAT, iWAT, MG and MS in treated and untreated mice. TA-1887 treatment remarkably decreased levels of p16^INK4a^ transcripts in eWAT, where expression of multiple inflammatory mediators was most markedly decreased, although p16^INK4a^ expression was comparable in treated versus untreated animals in other tissues, and p21 expression was similar in all tissues analyzed (Fig. 3c and Supplementary Fig. 1c). Moreover, accumulation of senescent cells in eWAT, iWAT, and MG was confirmed by assessing senescence-associated beta-galactosidase activity using SPiDER beta-Gal staining.^21^ Beta-Gal staining decreased in eWAT from TA-1887-treated relative to untreated mice, but was equivalent in iWAT and MG from treated and untreated mice (Fig. 3d). These findings suggest that TA-1887 antagonizes cellular senescence in specific cell types.

### TA-1887 alleviates oxidative stress in *db/db* mice fed a HF diet

**Fig. 4:**
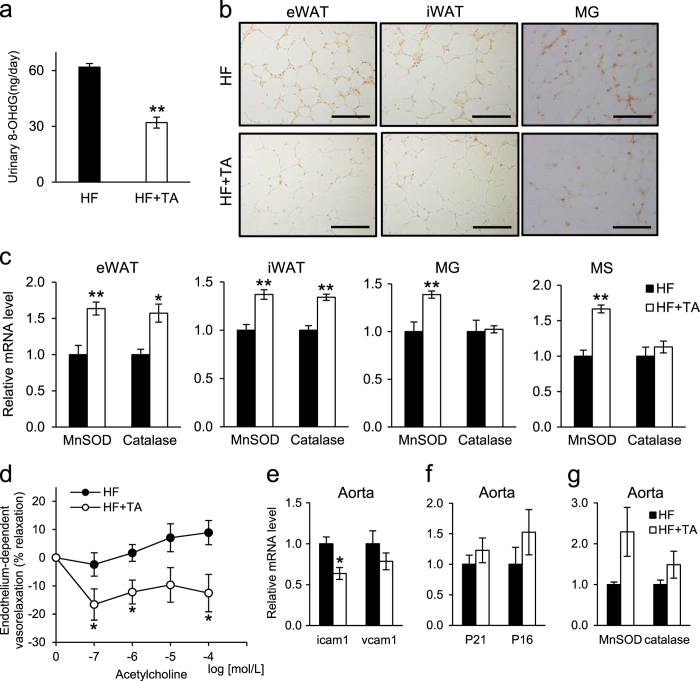
Effect of TA-1887 on urinary excretion and tissue expression of 8-OHdG, expression of antioxidative enzymes and vascular function in *db/db* mice fed a high-fat diet. **a** Urinary excretion of 8-OHdG in *db/db* mice fed a high-fat (HF) diet and treated for 4 months as indicated, as measured by ELISA (*n* = 5). **b** Immunohistochemistry with an 8-OHdG antibody of representative eWAT, iWAT and MG samples of mice treated 4 months as indicated. Scale bars: 200 μm (eWAT, iWAT), 100 μm (MG). **c** levels of mRNAs encoding the antioxidative enzymes MnSOD and catalase in eWAT, iWAT, MG and MS after 4 months of treatment (*n* = 5–9). **d** Endothelium-dependent vasorelaxation in response to acetylcholine in aorta after 4 months of treatment (*n* = 5–9). **e** Expression of transcripts associated with vascular inflammation in aorta of mice treated as indicated for 4 months (*n* = 5–9). **f** Expression of senescence marker transcripts in aorta after 4 months of indicated treatment (*n* = 5–9). **g** Expression of transcripts encoding antioxidative enzymes in aorta after 4 months of indicated treatment (*n* = 5–9). Values shown are means ± SEM. *p < 0.05 and **p < 0.01 versus HF

Oxidative stress impacts insulin resistance and senescence.^22, 23^ Thus we asked whether TA-1887 treatment modulated oxidative stress, as marked by 8-OHdG expression.^24^ TA-1887-treated *db/db* mice fed a HF diet showed decreased levels of urinary 8-OHdG relative to untreated mice, suggestive of decreased systemic oxidative stress (Fig. 4a). We then undertook immunostaining to detect 8-OHdG in eWAT, iWAT, and MG. TA-1887 treatment reduced 8-OHdG expression relative to untreated controls in all three tissues (Fig. 4b). Analysis of those tissues plus MS also showed that transcripts encoding the antioxidative enzymes Mn-SOD and catalase increased in samples from TA-1887-treated relative to untreated mice (Fig. 4c). Although we observed no difference in 8-OHdG immnostaining in BAT and liver from treated and untreated mice, catalase expression increased in liver of TA-1887-treated relative to untreated mice (Supplementary Fig. 1d). Increased expression of antioxidative enzymes, particularly in eWAT and iWAT, suggests that these factors may mediate reduced oxidative stress seen in response to TA-1887 treatment.

### TA-1887 improves endothelial function in *db/db* mice fed a high fat-diet

We next asked what effect TA-1887 treatment had on cardiovascular function of *db/db* mice fed a HF diet. To do so, we evaluated endothelium-dependent relaxation in response to acetylcholine^25, 26^ with or without drug. TA-1887-treated mice showed a slight relaxation response to acetylcholine, while that response was absent in the aorta of untreated mice (Fig. 4d). To address underlying mechanisms, we assessed expression of mRNAs encoding senescence markers or antioxidative enzymes in aorta tissue but observed no differences between groups (Fig. 4f, g). We then examined expression of genes encoding vascular inflammatory markers. Levels of transcripts encoding intracellular adhesion molecule-1 (Icam-1) decreased in TA-1887-treated relative to untreated groups, while those of vascular cell adhesion molecule-1 (Vcam-1) were comparable between groups (Fig. 4e).

### Insulin treatment increases fat volume but preserves pancreatic beta cell function and enhances glucose utilization

**Fig. 5:**
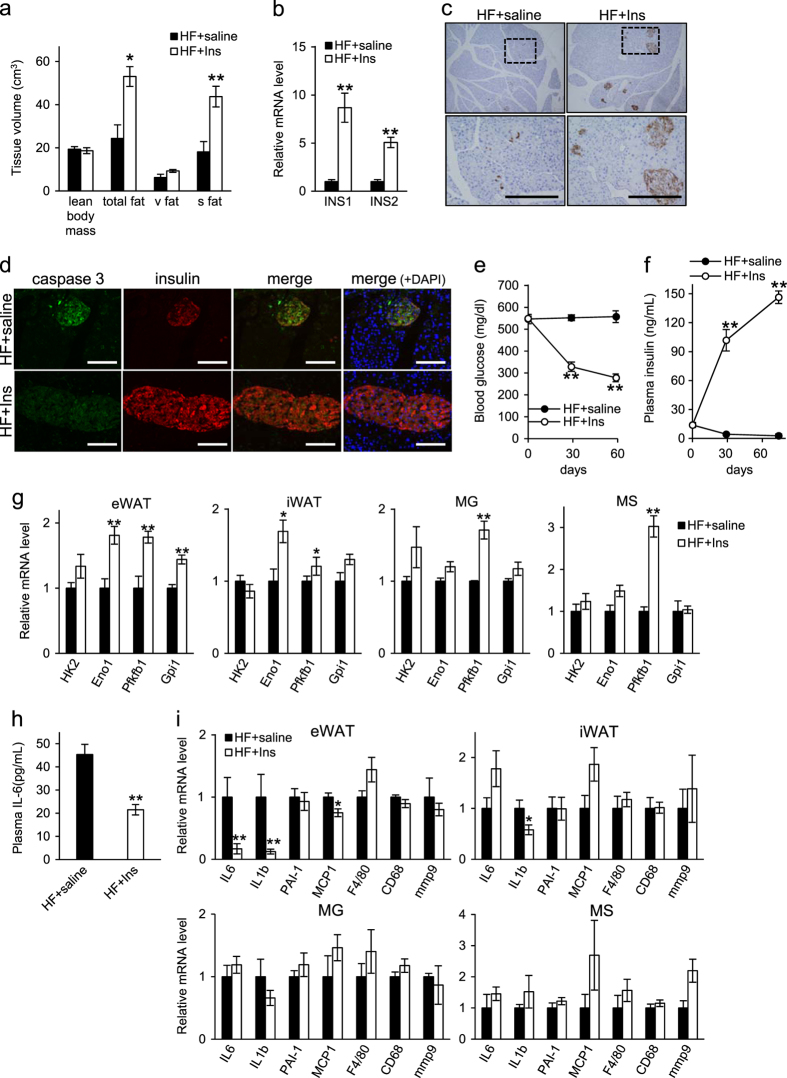
Effects of insulin on tissue composition, pancreatic beta cell, glucose metabolism and inflammation in *db/db* mice fed a high-fat (HF) diet. **a** Volume of lean body mass, total fat, visceral fat (v fat) and subcutaneous fat (s fat), as measured by Computed Tomography after 3 months of indicated treatment (*n* = 5). **b** Analysis of INS1 and INS2 mRNAs in samples of pancreatic tissue after 4 months of indicated treatment (*n* = 5–7). **c** Immunostaining for insulin in representative pancreatic tissue samples after 4 months of indicated treatment. Squares in upper panels are magnified in corresponding lower panels. Scale bars: 200 μm. **d** Double immunostaining for insulin and active caspase 3 in representative pancreatic tissues after 4 months of treatment. Scale bars: 100 μm. **e**, **f** Blood glucose and plasma insulin levels at 0, 1, and 2 months of drug treatment (*n* = 8). **g** levels of mRNAs encoding glycolytic enzymes in eWAT, iWAT, MG and MS after 4 months of indicated treatment (*n* = 5–7). **h** Plasma IL-6 concentrations as measured by ELISA after 2 months of drug treatment (*n* = 8). **i** levels of inflammatory mRNAs in eWAT, iWAT, MG and MS after 4 months of indicated treatment (*n* = 5–7). DAPI, 4',6-diamidino-2-phenylindole; INS1, insulin I; INS2, insulin II; HK2, hexokinase 2; Eno1, enolase 1; PFKFB1, 6-phosphofructo-2-kinase/fructose-2,6-biphosphatase 1; Gpi1, glucose phosphate isomerase 1; IL-6, interleukin-6; IL-1b, interleukin-1b; PAI-1, plasminogen activator inhibitor-1; MCP1, monocyte chemoattractant protein-1; mmp9, matrix metalloproteinase 9. Values shown are means ± SEM. *p < 0.05 and **p < 0.01 versus HF + saline

Given that insulin treatment of *db/db* mice fed a high fat-diet antagonizes diabetic cachexia and mortality (Fig. 1), we asked how insulin exerts this effect. CT scanning of insulin-treated mice revealed increased amounts of subcutaneous fat relative to untreated *db/db* mice fed a high fat-diet (Fig. 5a). Pancreatic tissue of insulin-treated mice also showed upregulated expression of INS1 and INS2 mRNAs relative to controls (Fig. 5b). Moreover, immunohistochemistry confirmed that pancreatic beta cell volume increased in insulin-treated mice, indicative of preserved pancreatic beta cell function (Fig. 5c). In addition, double immunostaining of insulin and active caspase 3 showed increased insulin staining but decreased staining of active caspase 3 in pancreatic islets of insulin-treated relative to untreated control mice, indicating that insulin treatment prevents beta cell death (Fig. 5d). Mice treated with insulin showed markedly increased plasma insulin levels and reduced blood glucose levels compared with untreated controls (Fig. 5e, f). Finally, some tissues (namely, eWAT, iWAT, MG and MS) of insulin-treated mice showed enhanced expression of mRNAs encoding glycolytic enzymes, suggesting that glucose utilization is enhanced in these organs (Fig. 5g).

### Insulin treatment has diverse effects on target organ gene expression and pathological state

We next evaluated gene expression and related pathological changes in peripheral insulin-sensitive organs of mice in the presence or absence of insulin treatment. Relevant to inflammatory markers, we observed that plasma IL-6 levels decreased in insulin-treated compared to untreated mice (Fig. 5h). In eWAT and iWAT, levels of mRNAs encoding inflammatory mediators decreased in insulin-treated relative to control mice, although levels were comparable between groups in MG and MS (Fig. 5i). Moreover, immunostaining for Mac-3 revealed reduced macrophage infiltration into eWAT and iWAT of insulin-treated mice relative to untreated controls (Supplementary Fig. 2b).

**Fig. 6:**
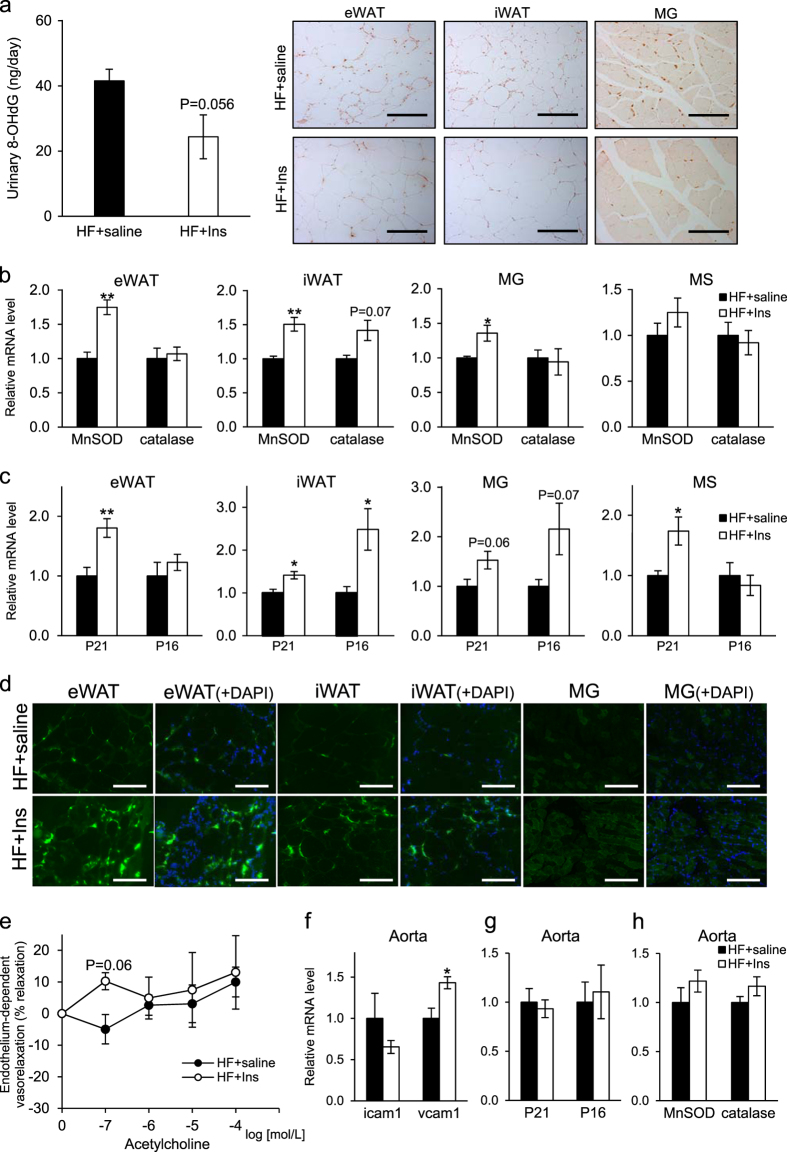
Effects of insulin treatment on senescence and oxidative stress markers and on vascular function in *db/db* mice fed a high-fat (HF) diet. **a** left: 8-OHdG concentration in urine as determined by ELISA after 4 months of treatment. right: Immunohistochemistry with an 8-OHdG antibody of representative eWAT, iWAT and MG samples after 4 months of indicated treatment. Scale bars: 200 μm (eWAT, iWAT), 100 μm (MG). **b** Levels of transcripts encoding the antioxidative enzymes MnSOD or catalase in mouse eWAT, iWAT, MG and MS following 4 months of indicated treatments (*n* = 5–7). **c** mRNA levels of the senescence markers p21 and p16^INK4a^ in eWAT, iWAT, MG and MS after 4 months of indicated treatment (*n* = 5–7). **d** Senescence-associated staining for SPiDER beta-Gal in frozen sections of eWAT, iWAT and MG tissues after 4 months of indicated treatment. Scale bars: 200 μm. **e** Endothelium-dependent vasorelaxation in response to acetylcholine in aorta after 4 months of treatment (*n* = 5–7). **f** Expression of transcripts associated with vascular inflammation in aorta after 4 months of treatment (*n* = 5–7). **g** Expression of transcripts of senescence markers in aorta after 4 months of indicated treatment (*n* = 5–7). **h** Expression of transcripts encoding antioxidative enzymes in aorta after 4 months of indicated treatment (*n* = 5–7). Values shown are means ± SEM. *p < 0.05 and **p < 0.01 versus HF + saline

In terms of oxidative stress, insulin-treated mice showed decreased urinary 8-OHdG levels relative to untreated mice and weaker immnohistochemical staining of 8-OHdG in eWAT, iWAT and MG relative to untreated controls (Fig. 6a). Moreover, expression of transcripts encoding the antioxidative enzyme Mn-SOD increased in eWAT, iWAT, and MG tissues of insulin-treated mice (Fig. 6b).

Finally, relevant to senescence markers, we found that insulin-treated mice showed increased expression of p21 mRNA in eWAT, iWAT, MG (*p* = 0.06) and MS, and of p16^INK4a^ mRNA in iWAT and MG (*p* = 0.07) relative to untreated mice (Fig. 6c). Consistent with these results, senescence-associated beta-Gal activity increased in eWAT, iWAT and MG from insulin-treated compared with untreated mice (Fig. 6d).

### Endothelial dysfunction is exacerbated by insulin treatment in *db/db* mice fed a HF diet

Finally, to assess effects of insulin on cardiovascular outcomes, we evaluated endothelial function in insulin-treated and control *db/db* mice fed a HF diet. Aorta tissue of insulin-treated mice did not exhibit any relaxation response to acetylcholine but rather showed an increased contractile response relative to the untreated group (Fig. 6e). Expression of mRNAs encoding p21 and p16 (Fig. 6g) and MnSOD and catalase (Fig. 6h) was comparable in aorta of insulin-treated and untreated groups; however, levels of Vcam-1 transcripts increased in aorta of insulin-treated relative to untreated groups, whereas Icam-1 levels were equivalent in both groups (Fig. 6f).

## Discussion

Here we have conducted parallel investigations of TA-1887 and insulin on diabetic complications in *db/db* mice fed a HF diet. We show overall that both TA-1887 and insulin decrease inflammation and oxidative stress, and preserve function of pancreatic beta cells and insulin target organs in these mice. Moreover, we find that in some cases TA-1887 may have more potent effects on endothelial function, cellular senescence and survival (see Supplementary Table 2 and Figs. 3–5).

We observe that TA-1887 treatment of *db/db* mice fed a HF diet enabled mice to gain body weight over time, preventing a cachectic state brought on by severe diabetes and decreasing mortality relative to untreated controls. Nonetheless, TA-1887-treated mice were obese and showed increased visceral and subcutaneous WAT. Exacerbation of obesity, particularly an increased visceral WAT, generally induces invasion of inflammatory cells, such as macrophages, and initiates adipose tissue inflammation followed by insulin resistance and aggravation of hyperglycemia.^27, 28^ Expression of inflammatory markers and macrophage infiltration, however, decreased in adipose tissue of TA-1887-treated mice compared with controls, and TA-1887-treated mice demonstrated increased insulin sensitivity and enhanced glucose utilization.

Visceral adipose tissue of TA-1887-treated mice also showed decreased expression of the senescence marker p16^INK4a^ relative to untreated mice. TA-1887-treated mice also showed decreased oxidative stress, which impacts insulin resistance and senescence, as indicated by marker analysis of urine and tissues, potentially due to increased expression of the antioxidative enzymes MnSOD and catalase.

Insulin secretion and enhanced insulin sensitivity is critical to avoid pathological weight loss and to store energy in adipose tissue.^29^ However, sustained hyperglycemia that accompanies severe obesity exhausts pancreatic beta cells, inducing their apoptosis and decreasing insulin secretion.^30, 31^ While untreated *db/db* mice fed a HF diet showed significantly decreased endogenous insulin levels (Fig. 2b), plasma insulin levels in TA-1887-treated mice were relatively stable, as was beta cell mass, possibly due to loss of glucotoxicity via increased urinary glucose excretion.

Insulin-treated diabetic mice also showed preserved pancreatic beta cell function (Fig. 5b, c). However, it is noteworthy that plasma insulin levels were 10-fold higher in insulin-relative to TA-1887-treated mice (compare values in Fig. 2b to those shown in Fig. 5f). Insulin signaling not only mediates glucose uptake and serves as a growth signal but is also involved in aging.^32, 33^ Accordingly, insulin-treated mice are exposed to higher levels of insulin than are TA-1887-treated mice, potentially accelerating cellular and tissue senescence. Hyperglycemia itself induces senescence through reactive oxygen species (ROS) production and advanced glycation end products.^34–36^ Hyperglycemia also induces macrophage infiltration in some organ tissues and escalates inflammatory conditions.^37^ Inflammatory mediators also induce senescence, and senescent cells produce senescence-associated secretory phenotypes factors, which initiate and propagate similar phenotypes in other cells.^38^ Insulin-treated mice showed reduced blood glucose and attenuated inflammation and oxidative stress but increased expression of senescence markers in all tissues analyzed, suggesting, that in this case, hyperinsulinemia (which was 10-fold higher than that seen in the TA-1887-treated group) is primarily responsible for senescence. High insulin concentrations can also activate the insulin-like growth factor-1 (IGF-1) receptor,^39^ and insulin/IGF-1 signaling induces ROS and promotes cellular senescence via the ROS-p53 pathway.^40, 41^ Insulin /IGF-1 signaling also reportedly promotes senescence phenotypes in the absence of inflammation or oxidative stress via several mechanisms. Among these are the p53-p21 pathway via PI3K,^42^ increased p53 stabilization and activation through SIRT1 inhibition,^43^ and ERK activation, which also upregulates p53 and promotes its stability and activity.^44, 45^ By contrast, TA-1887-treated mice did not show increased expression of senescence markers and in fact exhibited decreased p16^INK4a^ expression in visceral WAT. TA-1887 treatment also decreased blood glucose levels, inflammation and oxidative stress. We conclude that maintenance of appropriate blood glucose and insulin levels may antagonize senescence.

Previous reports suggest that adipose tissue is important in terms of survival.^46–48^ Decreased insulin/IGF-1 signaling in adipose tissue extends lifespan in *Drosophila* and mice,^46, 47^ and subsequent activation of the forkhead transcription factor (FOXO) may underlie longevity.^46, 48^ Interestingly, in eWAT and iWAT of TA-1887-treated mice, we observed increased expression of forkhead targets,^49^ such as genes that encode the antioxidative enzymes MnSOD and catalase, and relief of oxidative stress (Fig. 4a–c).

Hyperglycemia reportedly promotes vascular inflammation and endothelial dysfunction and contributes to vascular disease.^50^ Although TA-1887 or insulin treatment ameliorated hyperglycemia in diabetic mice, only TA-1887 attenuated endothelial dysfunction (Fig. 4d, 6e). Hyperinsulinemia-induced excess insulin activity caused by insulin administration promotes vascular inflammation by producing proinflammatory cytokines in vascular smooth muscle cells.^51^ It is also noteworthy that TA-1887 treatment decreased levels of Icam-1, but not of Vcam-1, while insulin treatment had the opposite effect, increasing Vcam-1 but not Icam-1 levels (Fig. 4e, 6f). Others have reported that high glucose stimulation upregulates Icam-1 but not Vcam-1 expression.^52, 53^ Furthermore, insulin stimulation reportedly promotes both Vcam-1 and Icam-1 expression in endothelial cells,^54^ supporting the idea that regulation of these factors differs. Taken together, differential effects of TA-1887 and insulin treatment on endothelial function may be due in part to differences in vascular inflammation caused by hyperinsulinemia as blood glucose levels improve, an event with consequences for mortality.

There is some concern that SGLT2 inhibitors, which activate gluconeogenesis, may induce muscle atrophy.^55, 56^ Our CT scan findings showed no reduction in lean body mass but rather the opposite tendency (Fig. 1f). Sano et al. reported that patients with type 2 diabetes treated with a SGLT2 inhibitor exhibit increased grip strength, indicating that SGLT2i treatment does not necessarily promote muscle weakness, a typical symptom of sarcopenia, but rather strengthens it.^57^ Long-term use of SGLT2i could rescue fat and glycogen synthesis and energy storage in skeletal muscle by improving insulin sensitivity and preserving endogenous insulin secretion, an effect that might antagonize increased lipolysis or muscle catabolism.

We also assessed vascular events related to type 2 diabetes and observed no sign of macrovascular events, such as brain hemorrhage, cerebral infarction or necrotic changes in myocardium (data not shown). Relevant to microvascular events, we evaluated proteinuria, a major complication of diabetes. We observed reduced proteinuria in both TA-1887-treated and insulin-treated versus untreated mice (Supplementary Fig. 6), supporting the idea that both TA-1887 and insulin treatments antagonize type 2 diabetes.

Finally, there are currently many treatment options for type 2 diabetes, and appropriate selection of therapy individualized to each patient is needed. To date, anti-diabetic agents with a hypoglycemic effect potent enough to relieve glucotoxicity, improve insulin sensitivity, and preserve endogenous insulin secretion with minimum load on pancreatic beta cells do not exist. SGLT2i, hence, could present an effective alternative treatment for type 2 diabetes, while potentially associated obesity could be prevented by appropriate dietary management. Further studies are required to explore this possibility.

## Materials and methods

### Materials

TA-1887 (3-(4-cyclopropylbenzyl)-4-fluoroindole-*N*-glucoside) was supplied by Mitsubishi Tanabe Pharma Corporation (Osaka, Japan).

### Animals

Six-week-old male *db/db* mice were purchased from CLEA Japan Inc (Tokyo, Japan). Mice were maintained in a pathogen-free facility under controlled environmental conditions and exposed to a 12:12 h light:dark cycle. After 2 weeks of acclimation, mice were fed HF diets (HFD-32; CLEA Japan Inc., Tokyo, Japan) with or without TA-1887 treatment (0.01% w/w in chow). To assess effects of chronic insulin treatment, animals attached to either insulin or normal saline pumps (Alzet, model 2002; DURECT, Cupertino, CA) were similarly fed and received insulin (3 μg/g/day) or control saline, respectively. Blood glucose levels of insulin-treated mice were adjusted to ~200 mg/dl by additional administration of long-acting insulin (Insulin Glargine, Sanofi, Gentilly, France). Animal experiments were approved by the institutional review board at Kumamoto University, and all animals received humane care.

### Indirect calorimetry

Energy expenditure was measured using an indirect calorimetry system (MK-5000RQ, Muromachi Kikai Co., Ltd., Tokyo, Japan), as previously reported.^58^

### Computed tomography (CT)

Mice were anesthetized by intraperitoneal injection of pentobarbital, and adiposity was assessed using an X-ray CT system (La Theta; Aloka Ltd., Tokyo, Japan).

### Survival analysis

Eight-week-old male *db/db* mice fed a HF diet were assigned to four groups: high-fat (*n* = 30), high-fat with TA-1887 (*n* = 30), saline pump (*n* = 20), and insulin pump (*n* = 20) groups for survival analysis. Survival was monitored several times a week. Survival curves were plotted using the Kaplan Meier method.

### Intraperitoneal insulin tolerance test (IPITT)

After 10 weeks on each diet, mice fasted overnight (14 h) underwent IPITT with a 0.75 U/kg body weight insulin solution. Tail vein blood glucose levels were determined using a STAT STRIP Xpress 900 monitor (Nova Biomedical Corporation, Waltham, MA).

### Enzyme-linked immunosorbent assay (ELISA)

Plasma insulin was assessed using the Morinaga Ultra-Sensitive Mouse/Rat Insulin ELISA Kit according to the manufacturer’s recommendations (Morinaga Institute of Biological Science, Inc., Yokohama, Japan). Plasma IL-6 concentrations were determined using Mouse IL-6 ELISA MAX Deluxe Sets (Biolegend, San Diego, CA). 8-hydroxy-2’-deoxyguanosine (8-OHdG) concentrations in urine were measured by ELISA (Nikken Seil, Shizuoka, Japan).

### Quantitative real-time PCR

Total RNA was extracted using TRIzol reagent according to the manufacturer’s protocol. DNase-treated RNA was reverse transcribed using a PrimeScript RT reagent Kit (Takara Bio Inc., Shiga, Japan). Quantitative real-time PCR was performed using SYBER Premix Ex Taq II (Takara Bio Inc.). Relative transcript abundance was normalized to that of 18 S rRNA levels. Primer sequences are shown in Supplementary Table 1.

### Immunostaining

For all procedures, samples were fixed in 4% paraformaldehyde for 24 h and embedded in paraffin blocks, which were cut into 4-μm sections, air-dried and then deparaffinized. For immunohistochemistry, after antigen retrieval endogenous peroxidase activity was blocked by treating sections with either 3% H_2_O_2_ in Tris-buffered saline for 10 min, or, in the case of 8-OHdG detection, 0.5% H_2_O_2_ in methanol for 30 min. Sections were then blocked with 5% goat serum for 20 min at room temperature (RT) and incubated with primary antibodies overnight at 4 °C. After PBS washing, sections were treated with secondary antibodies using Histofine Simple Stain MAX-PO (Nichirei Biosciences Inc., Tokyo, Japan) or an EnVision System-HRP kit (Dako, Carpinteria, CA), according to the manufacturers’ instructions. To detect 8-OHdG, blocking and secondary antibody reactions were carried out using a Histofine mouse staining kit (Nichirei Biosciences Inc., Tokyo, Japan). Peroxidase activity was visualized by incubation with a 3,3-diaminobenzidine solution. Slides were counterstained with hematoxylin and mounted. Antibodies used were: anti-insulin (1:100, sc-9168, Santa Cruz Bio, Dallas, TX), anti-8-OHdG (1:20, Nikken Seil, Shizuoka, Japan) and anti-Mac-3 (1:100, BD Biosciences, Franklin Lakes, NJ). For double immunofluorescence of pancreatic tissue, endogenous biotin and peroxidase activity was blocked using a Biotin Blocking System (Dako, Carpinteria, CA) and 3% H_2_O_2_, respectively. Sections were then incubated overnight with anti-active caspase 3 antibody (1:250, Promega Corp., Madison, WI), and staining performed using a Tyramide Signal Amplification kit (PerkinElmer, Boston, MA). After PBS washing, specimens were incubated with anti-insulin antibody (1:100, sc-9168, Santa Cruz Bio, Dallas, TX) overnight at 4 °C. After PBS washing, sections were incubated with Alexa Fluor 594-labeled anti-rabbit IgG (1:500, Invitrogen Corp., Carlsbad, CA) and Streptavidin-Fluorescein (1:500, PerkinElmer, Boston, MA) as second antibodies. Fluorescent imaging was performed after PBS washing.

### SPiDER beta-Gal staining

Tissues (eWAT, iWAT and MG) were placed in O.C.T. Compound (Sakura Finetek USA Inc., Torrance, CA) in Tissue-Tek Cryomolds (Sakura Finetek USA Inc., Torrance, CA) and flash-frozen in hexane cooled with solid carbon dioxide. Sections (WAT: 15 μm, MG: 6 μm) were cut using a cryostat, and mounted onto glass slides. They were then fixed in 4% paraformaldehyde for 20 min at RT, washed in PBS, and immersed in 20-μM SPiDER beta-Gal staining solution^21^ (Dojindo Molecular Technologies, Inc., Rockville, MD) for 1 h at 37 °C. Imaging was performed after washing with PBS.

### Vascular endothelial function

After mice were killed, the aorta was removed and measured for vascular endothelial function. Pressurized aortas were kept in a chamber of warmed (37 °C) and oxygenated (95% air-5% CO_2_) Krebs solution. Endothelium-dependent relaxation was assessed by measuring dilatory responses to increasing acetylcholine concentration (10^−7^–10^−4^ mol/L) in vessels pre-treated with phenylephrine at 5 × 10^−5^ mol/L.

### Statistical analyses

Results are reported as means ± standard error (SEM). Statistical differences were determined using the unpaired two-tailed Student’s *t*-test or Kruskal–Wallis tests with Bonferroni correction for multiple comparisons. Kaplan–Meier analysis was performed by the log-rank statistic with the Holm’s method to test for significant differences in survival. Statistical significance is reported as a *P* value < 0.05 or <0.01.

### Data availability

All data that support the findings of this study are in this published article and its Supplementary information, or are available from the corresponding author on reasonable request.

## Electronic supplementary material

Supplementary information

## Electronic supplementary material

**Supplementary Information** accompanies the paper on the *npj Aging and Mechanisms of Disease* website (10.1038/s41514-017-0012-0).

## References

[1] Stratton IM (2000). Association of glycaemia with macrovascular and microvascular complications of type 2 diabetes (UKPDS 35): prospective observational study. BMJ.

[2] Emerging Risk Factors Collaboration. (2010). Diabetes mellitus, fasting blood glucose concentration, and risk of vascular disease: a collaborative meta-analysis of 102 prospective studies. Lancet.

[3] UK Prospective Diabetes Study (UKPDS (1998). Group Intensive blood-glucose control with sulphonylureas or insulin compared with conventional treatment and risk of complications in patients with type 2 diabetes (UKPDS 33). Lancet.

[4] UK Prospective Diabetes Study (UKPDS (1998). Group Effect of intensive blood-glucose control with metformin on complications in overweight patients with type 2 diabetes (UKPDS 34). Lancet.

[5] Holman RR (2008). 10-year follow-up of intensive glucose control in type 2 diabetes. N. Engl. J. Med..

[6] Group AC (2008). Intensive blood glucose control and vascular outcomes in patients with type 2 diabetes. N. Engl. J. Med..

[7] Zinman B (2015). Empagliflozin, cardiovascular outcomes, and mortality in type 2 diabetes. N. Engl. J. Med..

[8] Cardoso CR, Ferreira MT, Leite NC, Salles GF (2013). Prognostic impact of aortic stiffness in high-risk type 2 diabetic patients: the Rio deJaneiro type 2 diabetes cohort study. Diabetes Care.

[9] Bakris GL, Molitch M (2014). Microalbuminuria as a risk predictor in diabetes: the continuing saga. Diabetes Care.

[10] Tikkanen I (2015). Empagliflozin reduces blood pressure in patients with type 2 diabetes and hypertension. Diabetes Care.

[11] Nomura S (2014). Novel Indole-N-glucoside, TA-1887 as a sodium glucose cotransporter 2 inhibitor for treatment of type 2 diabetes. ACS Med Chem. Lett..

[12] Kuriyama C (2014). Analysis of the effect of canagliflozin on renal glucose reabsorption and progression of hyperglycemia in zucker diabetic Fatty rats. J. Pharmacol. Exp. Ther..

[13] Bolinder J (2012). Effects of dapagliflozin on body weight, total fat mass, and regional adipose tissue distribution in patients with type 2 diabetes mellitus with inadequate glycemic control on metformin. J. Clin. Endocrinol. Metab..

[14] Suzuki M (2014). Tofogliflozin, a sodium/glucose cotransporter 2 inhibitor, attenuates body weight gain and fat accumulation in diabetic and obese animal models. Nutr. Diabetes.

[15] Zhang HM (2012). Geldanamycin derivative ameliorates high fat diet-induced renal failure in diabetes. PLoS One.

[16] Okada-Iwabu M (2013). A small-molecule AdipoR agonist for type 2 diabetes and short life in obesity. Nature.

[17] Hotamisligil GS (2006). Inflammation and metabolic disorders. Nature.

[18] Shoelson SE, Lee J, Goldfine AB (2006). Inflammation and insulin resistance. J. Clin. Invest..

[19] Tominaga K (2015). The emerging role of senescent cells in tissue homeostasis and pathophysiology. Pathobiol. Aging Age Relat. Dis..

[20] Childs BG, Durik M, Baker DJ, van Deursen JM (2015). Cellular senescence in aging and age-related disease: from mechanisms to therapy. Nat. Med..

[21] Doura T (2016). Detection of LacZ-positive cells in living tissue with single-cell resolution. Angew. Chem. Int. Ed. Engl..

[22] Ceriello A, Motz E (2004). Is oxidative stress the pathogenic mechanism underlying insulin resistance, diabetes, and cardiovascular disease? The common soil hypothesis revisited. Arterioscler. Thromb. Vasc. Biol..

[23] Salmon, A. B. Beyond diabetes: does obesity-induced oxidative stress drive the aging process? *Antioxidants (Basel*) **5**, 24 (2016). 10.3390/antiox5030024 PMC5039573 27438860

[24] Valavanidis A, Vlachogianni T, Fiotakis C (2009). 8-hydroxy-2’ -deoxyguanosine (8-OHdG): A critical biomarker of oxidative stress and carcinogenesis. J. Environ. Sci. Health C Environ. Carcinog. Ecotoxicol. Rev..

[25] Hodgson JM, Marshall JJ (1989). Direct vasoconstriction and endothelium-dependent vasodilation. Mechanisms of acetylcholine effects on coronary flow and arterial diameter in patients with nonstenotic coronary arteries. Circulation.

[26] Matsubara J (2012). A dipeptidyl peptidase-4 inhibitor, des-fluoro-sitagliptin, improves endothelial function and reduces atherosclerotic lesion formation in apolipoprotein E-deficient mice. J. Am. Coll. Cardiol..

[27] Stienstra R (2011). Inflammasome is a central player in the induction of obesity and insulin resistance. Proc. Natl. Acad. Sci. USA.

[28] Kanda H (2006). MCP-1 contributes to macrophage infiltration into adipose tissue, insulin resistance, and hepatic steatosis in obesity. J. Clin. Invest..

[29] Raz I, Eldor R, Cernea S, Shafrir E (2005). Diabetes: insulin resistance and derangements in lipid metabolism. Cure through intervention in fat transport and storage. Diabetes Metab. Res. Rev..

[30] Wajchenberg BL (2007). beta-cell failure in diabetes and preservation by clinical treatment. Endocr. Rev..

[31] Guillausseau PJ (2008). Abnormalities in insulin secretion in type 2 diabetes mellitus. Diabetes Metab..

[32] Kimura KD, Tissenbaum HA, Liu Y, Ruvkun G (1997). daf-2, an insulin receptor-like gene that regulates longevity and diapause in Caenorhabditis elegans. Science.

[33] Clancy DJ (2001). Extension of life-span by loss of CHICO, a Drosophila insulin receptor substrate protein. Science.

[34] Blazer S (2002). High glucose-induced replicative senescence: point of no return and effect of telomerase. Biochem. Biophys. Res. Commun..

[35] Ksiazek K, Passos JF, Olijslagers S, von Zglinicki T (2008). Mitochondrial dysfunction is a possible cause of accelerated senescence of mesothelial cells exposed to high glucose. Biochem. Biophys. Res. Commun..

[36] Liu J (2014). Receptor for advanced glycation end-products promotes premature senescence of proximal tubular epithelial cells via activation of endoplasmic reticulum stress-dependent p21 signaling. Cell. Signal..

[37] Niu S (2016). Broad infiltration of macrophages leads to a proinflammatory state in streptozotocin-induced hyperglycemic mice. J. Immunol..

[38] Nelson G (2012). A senescent cell bystander effect: senescence-induced senescence. Aging Cell..

[39] Boucher J, Tseng YH, Kahn CR (2010). Insulin and insulin-like growth factor-1 receptors act as ligand-specific amplitude modulators of a common pathway regulating gene transcription. J. Biol. Chem..

[40] Katic M, Kahn CR (2005). The role of insulin and IGF-1 signaling in longevity. Cell. Mol. Life Sci..

[41] Handayaningsih AE (2012). IGF-I enhances cellular senescence via the reactive oxygen species-p53 pathway. Biochem. Biophys. Res. Commun..

[42] Clark MA, Perks CM, Winters ZE, Holly JM (2005). DNA damage uncouples the mitogenic response to IGF-I in MCF-7 malignant breast cancer cells by switching the roles of PI3 kinase and p21WAF1/Cip1. Int. J. Cancer.

[43] Tran D (2014). Insulin-like growth factor-1 regulates the SIRT1-p53 pathway in cellular senescence. Aging Cell.

[44] Nguyen TT, Sheppard AM, Kaye PL, Noakes PG (2007). IGF-I and insulin activate mitogen-activated protein kinase via the type 1 IGF receptor in mouse embryonic stem cells. Reproduction.

[45] Cagnol S, Chambard JC (2010). ERK and cell death: mechanisms of ERK-induced cell death--apoptosis, autophagy and senescence. FEBS J..

[46] Hwangbo DS (2004). Drosophila dFOXO controls lifespan and regulates insulin signalling in brain and fat body. Nature.

[47] Bluher M, Kahn BB, Kahn CR (2003). Extended longevity in mice lacking the insulin receptor in adipose tissue. Science.

[48] Giannakou ME (2004). Long-lived Drosophila with overexpressed dFOXO in adult fat body. Science.

[49] Ponugoti B, Dong G, Graves DT (2012). Role of forkhead transcription factors in diabetes-induced oxidative stress. Exp. Diabetes Res..

[50] Bakker W, Eringa EC, Sipkema P, van Hinsbergh VW (2009). Endothelial dysfunction and diabetes: roles of hyperglycemia, impaired insulin signaling and obesity. Cell. Tissue Res..

[51] Sato Y (2007). Increased expression of CCAAT/enhancer binding protein-beta and -delta and monocyte chemoattractant protein-1 genes in aortas from hyperinsulinaemic rats. Diabetologia.

[52] Park CW (2000). High glucose-induced intercellular adhesion molecule-1 (ICAM-1) expression through an osmotic effect in rat mesangial cells is PKC-NF-kappa B-dependent. Diabetologia.

[53] Baumgartner-Parzer SM (1995). Modulation by high glucose of adhesion molecule expression in cultured endothelial cells. Diabetologia.

[54] Li G (2009). Insulin and insulin-like growth factor-I receptors differentially mediate insulin-stimulated adhesion molecule production by endothelial cells. Endocrinology.

[55] Kuo T, Harris CA, Wang JC (2013). Metabolic functions of glucocorticoid receptor in skeletal muscle. Mol. Cell. Endocrinol..

[56] Sandri M (2010). Autophagy in skeletal muscle. FEBS Lett..

[57] Sano, M., Meguro S., Kawai T., Suzuki Y. Increased grip strength with sodium-glucose cotransporter 2 inhibitors. *J. Diabetes* **8**, 736–737 (2016). 10.1111/1753-0407.12402 27038414

[58] Kouyama R (2005). Attenuation of diet-induced weight gain and adiposity through increased energy expenditure in mice lacking angiotensin II type 1a receptor. Endocrinology.

