# Supplementary material for: Treatment of diabetic mice with the SGLT2 inhibitor TA-1887 antagonizes diabetic cachexia and decreases mortality
Source: NPJ Aging Mech Dis. 2017 Sep 8;3:12. doi: 10.1038/s41514-017-0012-0 (PMC5591191; doi:10.1038/s41514-017-0012-0)
Supplement: Supplementary file 1 — Supplementary information [file 41514_2017_12_MOESM1_ESM.docx]

**Supplementary information**

**Supplementary Table 1: Primer sequences used in Real-time PCR**

| Gene |  | Sequences (5'-3') |
| --- | --- | --- |
| 18S | for | TTCTGGCCAACGGTCTAGACAAC |
|  | rev | CCAGTGGTCTTGGTGTGCTGA |
| INS1 | for | GACCATCAGCAAGCAGGTCA |
|  | rev | CTCCCAGAGGGCAAGCAG |
| INS2 | for | GCTTCTTCTACACACCCATGTC |
|  | rev | AGCACTGATCTACAATGCCAC |
| HK2 | for | GAGAACCGTGGACTGGACAA |
|  | rev | CCAGGAAGGACACGTCACAT |
| Eno1 | for | TGCGTCCACTGGCATCTAC |
|  | rev | CAGAGCAGGCGCAATAGTTTTA |
| Pfkfb1 | for | ATGAGCTGCCCTATCTCAAGT |
|  | rev | GTCCCGGTGTGTGTTCACAG |
| Gpi1 | for | CGGAAAGGTCTGCATCACAA |
|  | rev | CCTTCATCAGGGCCTCAGTC |
| IL-6 | for | CCACTTCACAAGTCGGAGGCTTA |
|  | rev | GCAAGTGCATCATCGTTGTTCATAC |
| IL-1b | for | TCCAGGATGAGGACATGAGCAC |
|  | rev | GAACGTCACACACCAGCAGGTTA |
| PAI-1 | for | TTCCAAGGCATCCAGAAGCAG |
|  | rev | CCGGAAATGACACATTGAAGTGAG |
| MCP1 | for | CCCAATGAGTAGGCTGGAGA |
|  | rev | GCTGAAGACCTTAGGGCAGA |
| F4/80 | for | GAGATTGTGGAAGCATCCGAGAC |
|  | rev | GATGACTGTACCCACATGGCTGA |
| Cd68 | for | CATCAGAGCCCGAGTACAGTCTACC |
|  | rev | AATTCTGCGCCATGAATGTCC |
| MMP-9 | for | GCCCTGGAACTCACACGACA |
|  | rev | TTGGAAACTCACACGCCAGAAG |
| p16^Ink4a^ | for | CGAACTCTTTCGGTCGTACCC |
|  | rev | CGAATCTGCACCGTAGTTGAGC |
| P21 | for | TCAGAGCCACAGGCACCTA |
|  | rev | TCCACGGGACCGAAGAGA |
| Mn-SOD | for | TCGCTTACAGATTGCTGCCT |
|  | rev | **C**GTGCTCCCACACGTCAATC |
| catalase | for | CCAGCGACCAGATGAAGCAG |
|  | rev | CCACTCTCTCAGGAATCCGC |
| Icam1 | for | CAATTCACACTGAATGCCAGCTC |
|  | rev | CAAGCAGTCCGTCTCGTCCA |
| Vcam1 | for | TGCCGGCATATACGAGTGTGA |
|  | rev | CCCGATGGCAGGTATTACCAAG |

**Supplementary Table 2: Study summary.**

Table indicates changes relative to controls after each treatment.

**
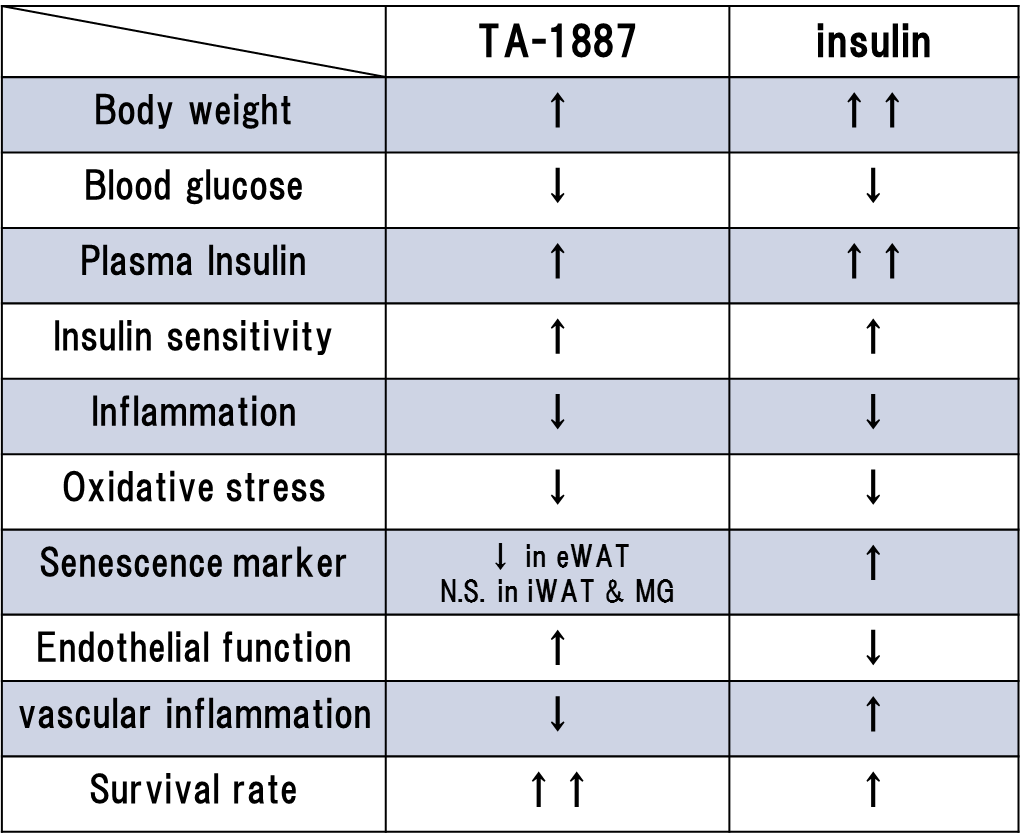
**

*** N.S., no significant difference between treated and untreated controls.**

**Supplementary Table 3: Assessment of cause of death in treated and untreated mice.**

**Supplementary Figure 1.**

**Marker expression and 8-OHdG immunostaining in BAT and liver of *db/db* mice fed a high-fat (HF) diet and treated for 4 months with TA-1887.**

(a) levels of transcripts encoding glycolytic enzymes in BAT (n=5-9).

(b) levels of inflammatory transcripts in BAT and liver (n=5-9).

(c) levels of transcripts encoding senescence markers in BAT and Liver (n=5-9).

(d) Left; Immunostaining for 8-OHdG in representative BAT and Liver samples of indicated treatment. Scale bar: 200 μm. Right; levels of mRNAs encoding anti-oxidative enzymes in BAT and Liver (n=5-9).


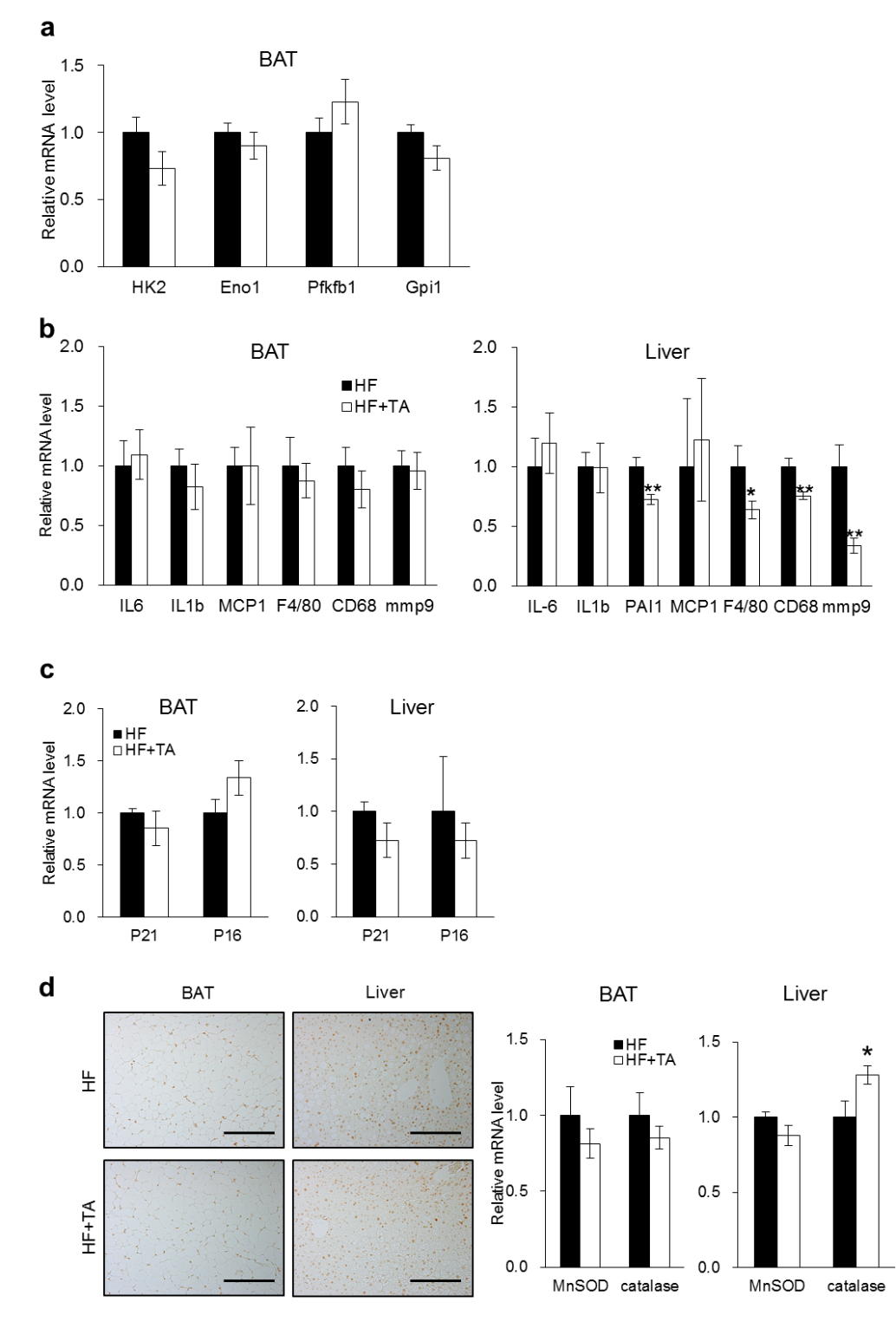
Values shown are means ± SEM. *p<0.05 and ***p*<0.01 vs HF

**Supplementary Figure 2.**

**Mac-3 immunostaining in eWAT and iWAT of *db/db* mice fed a high-fat (HF) diet and treated for 4 months with TA-1887 or insulin.**

(a) Immunostaining for Mac-3 in eWAT and iWAT of TA-1887-treated or untreated mice. Scale bar: 200 μm

(b) Immunostaining for Mac-3 in eWAT and iWAT of insulin-treated or untreated mice. Scale bar: 200 μm,


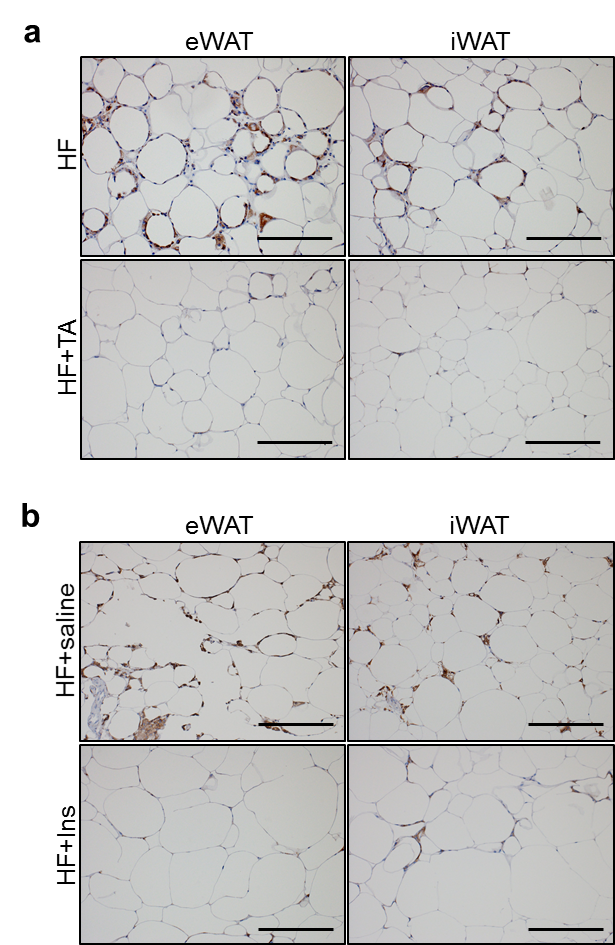


**Supplementary Figure 3.**

**Combined data from various treatment studies.**

(a,b) Blood glucose and plasma insulin (n=8) levels at 0, 1, and 2 months of indicated treatments.

(c) Volume of lean body mass, total fat, visceral fat (v fat) and subcutaneous fat (s fat), as measured by Computed Tomography (CT) after 3 months of indicated treatment (n=5-6).

(d) Plasma IL-6 concentration after 2 months of indicated treatments, as determined by ELISA (n=8).

(e) Urinary excretion of 8-OHdG in *db/db* mice fed a high-fat diet and treated 4 months as indicated, as measured by ELISA (n=5).

(f) Endothelium-dependent vasorelaxation in response to acetylcholine in aorta after 4 months of various treatment (n=5-9).

Values shown are means ± SEM. *p<0.05 and ***p*<0.01 vs relevant controls.

HF, high-fat diet; HF+TA, HF diet plus TA-1887 treatment; HF+saline, HF diet plus saline treatment; HF+Ins, HF diet plus insulin treatment.

**Supplementary Figure 4.**

**Combined gene expression data following indicated treatments.**

(a) levels of mRNAs encoding glycolytic enzymes in eWAT, iWAT, MG and MS following 4 months of indicated treatment (n=5-9).

(b) levels of mRNA encoding inflammatory mediators in eWAT, iWAT, MG and MS after 4 months of in indicated treatment (n=5-9).

Values shown are means ± SEM. *p<0.05 and ***p*<0.01 vs relevant controls.

HF, high-fat diet; HF+TA, HF diet plus TA-1887 treatment; HF+saline, HF diet plus saline treatment; HF+Ins, HF diet plus insulin treatment.

**Supplementary Figure 5.**

**Combined gene expression data following indicated treatments.**

(a) levels of mRNAs encoding the senescence markers p21 and p16^INK4a^ in eWAT, iWAT, MG and MS after 4 months of indicated treatment (n=5-9).

(b) levels of mRNAs encoding the antioxidative enzymes MnSOD and catalase in eWAT, iWAT, MG and MS after 4 months of indicated treatment (n=5-9).

(c) Expression of transcripts associated with vascular inflammation in aorta after 4 months of indicated treatment (n=5-9).

Values shown are means ± SEM. *p<0.05 and ***p*<0.01 vs relevant controls.

HF, high-fat diet; HF+TA, HF diet plus TA-1887 treatment; HF+saline, HF diet plus saline treatment; HF+Ins, HF diet plus insulin treatment.

**Supplementary Figure 6.**

**Urinary protein-to-creatinine ratio in various treatment groups.**

Ratios are calculated in *db/db* mice fed a high-fat (HF) diet and treated 4 months with TA-1887 or insulin. (n=5).

Values shown are means ± SEM. *p<0.05 and **p<0.01 versus relevant controls.
